# Musashi-2 Silencing Exerts Potent Activity against Acute Myeloid Leukemia and Enhances Chemosensitivity to Daunorubicin

**DOI:** 10.1371/journal.pone.0136484

**Published:** 2015-08-26

**Authors:** Yixiang Han, Aifang Ye, Yan Zhang, Zhimin Cai, Wei Wang, Lan Sun, Songfu Jiang, Jianbo Wu, Kang Yu, Shenghui Zhang

**Affiliations:** 1 Laboratory of Internal Medicine, the First Affiliated Hospital of Wenzhou Medical University, Wenzhou, 325015, China; 2 Key Laboratory of Molecular Medicine, Ministry of Education, and Department of Biochemistry and Molecular Biology, Fudan University Shanghai Medical College, Shanghai, 200032, China; 3 Department of Hematology, the First Affiliated Hospital of Wenzhou Medical University, Wenzhou 325015, China; University of Turin, ITALY

## Abstract

RNA-binding protein Musashi-2 (Msi2) is known to play a critical role in leukemogenesis and contributes to poor clinical prognosis in acute myeloid leukemia (AML). However, the effect of Msi2 silencing on treatment for AML still remains poorly understood. In this study, we used lentivirus-mediated RNA interference targeting Msi2 to investigate the resulting changes in cellular processes and the underlying mechanisms in AML cell lines as well as primary AML cells isolated from AML patients. We found that Msi2 was highly expressed in AML cells, and its depletion inhibited Ki-67 expression and resulted in decreased in vitro and in vivo proliferation. Msi2 silencing induced cell cycle arrest in G0/G1 phase, with decreased Cyclin D1 and increased p21 expression. Msi2 silencing induced apoptosis through down-regulation of Bcl-2 expression and up-regulation of Bax expression. Suppression of Akt, Erk1/2 and p38 phosphorylation also contributed to apoptosis mediated by Msi2 silencing. Finally, Msi2 silencing in AML cells also enhanced their chemosensitivity to daunorubicin. Conclusively, our data suggest that Msi2 is a promising target for gene therapy to optimize conventional chemotherapeutics in AML treatment.

## Introduction

Acute myeloid leukemia (AML) is a group of clonal hematopoietic stem cell disorders characterized by failure to differentiation and overproliferation in the stem cell compartment leading to accumulation of myeloblasts. Refinements of supportive treatment have contributed to improved outcomes of AML patients in the past 30 years. However, more than half of young adult patients and approximately 90% of older patients still succumb to their diseases [1]. One major obstacle to cure is relapse after complete remission. Accumulating evidences have suggested that leukemic stem cells are key drivers of pathogenesis and relapse [2]. Therefore, potential gene therapy strategies for leukemic stem cells may provide a novel way to optimize AML therapy.

The Musashi (Msi) family is an evolutionarily conserved group of RNA-binding proteins containing two RNA recognition motifs and plays a critical role in cell fate determination, asymmetric stem cell division and stem cell function regulation [3–5]. In mammals, two homologues of the Msi protein, Msi1 and Msi2 have been identified. Msi1 binds to the 3’ untranslated regions (UTRs) of target mRNAs at a consensus sequence, competing with eukaryotic initiation factor-4G for poly-A-binding protein binding and then blocking translation by preventing the formation of the 80S ribosomes [6]. The expression of Msi1 was found to associate with aggressive behavior in several tumors [7,8]. In the hematopoietic system, Msi2 expression was much higher than Msi1, and was particularly elevated in stem cells [9]. Elevated expression of Msi2 has been associated with poor clinical prognosis in patients with AML, adult B-cell acute lymphoblastic leukemia, or hepatocellular carcinoma [10–15].

Although Msi2 has been regarded as a new prognostic marker in leukemia, Msi2 directly interacts with and retains efficient translation of crucial transcription factors and epigenetic modulators including HOXA9, IKZF2, and MYC so as to directly maintain the mixed-lineage leukemia self-renewal program [16]. Msi2 expression was found to be associated with up-regulation of the cell cycle genes Cyclin D1 and Cdk2 and the self-renewal agonists HoxA9 and HoxA10 [17]. Msi2 has been found to activate Notch signaling by binding to the mRNA of Numb and preventing its translation in a murine model of chronic myeloid leukemia (CML) [9]. However, the expression of Msi2 was associated with FLT3-ITD positive, NPM1 and DNMT3A mutated, but not NUMB expression in AML patients [14]. Therefore, further investigation needs to be carried out to clarify the underlying mechanisms of Msi2 in AML.

In the present study, we demonstrated that Msi2 silencing decreased proliferation, induced cell cycle arrest in G0/G1 phase, and increased apoptosis in AML cell line Dami, HL-60, and primary AML cells from patients with AML, which were attributed to suppression of Akt, Erk1/2 and p38 phosphorylation. Msi2 silencing in AML cells also increased their chemosensitivity to daunorubicin.

## Materials and Methods

### Cell lines and primary AML cells

Human AML cell lines HL-60, NB4, U937, HEL, and Dami and CML cell line K562 were maintained in RPMI1640 medium supplemented with 10% fetal bovine serum (FBS; Gibco, Grand island, NY, USA). Primary AML cells were obtained from bone marrow aspirates of 8 newly diagnosed and untreated patients with AML (M1, 4; M2, 2; M4, 2; the diagnosis and classification was established according to the French-America-British criteria) according to our previously described methods [18], which were mixed with a very small amount of lymphocytes. All protocols and experiments were approved by the First Affiliated Hospital of Wenzhou Medical University institutional review board for clinical experiments and use of human samples; written consents were obtained from all subjects participated in this study in accordance with the Declaration of Helsinki protocol.

### Lentiviral-mediated RNA interference

Msi2 silencing was carried out with four separate short hairpin RNA (shRNA) constructs targeting Msi2 (shMsi2-1, shMsi2-2, shMsi2-3, and shMsi2-4). One scramble sequence was used as a negative control. The shMsi2 sequences and the scramble sequence see Table 1. The sequence was cloned into the LV3 shuttle plasmid with a RSV and CMV and H1 three promoter-driven GFP expression cassette. The lentiviral expression construct and three packaging plasmids were co-transfected into 293T cells with RNAi-Mate reagent (GenePharma, Shanghai, China). Supernatants were collected 72 h after transfection and filtered. Dami cells, HL-60 cells, and primary AML cells were infected with lentivirus in the presence of 5 μg/mL polybrene (Sigma-Aldrich, St Louis, MO, USA). At 24 h post-infection, the medium was removed and replaced with fresh growth medium containing 1 μg/mL puromycin (Gibco) and subsequently selected for infected cells for 48 h.

**Table 1:** The shMsi2 sequences and the scramble sequence.

	sequence (5’-3’)
shMsi2-1	TTGGAGAAATTAGAGAATGTA
shMsi2-2	GACCCAGCAAGTGTAGATAAA
shMsi2-3	GTGGAAGATGTAAAGCAATAT
shMsi2-4	TTGCCGGTTTCACAAGACATA
scramble	TTCTCCGAACGTGTCACGT

### Cytotoxicity assay

AML cells untreated and treated with scramble or shMsi2 lentivirus were seeded at a density of 4 × 10^3^/well in 96-well plates for 1–3 days, and cell viability was assessed using Cell Counting Kit-8 (CCK-8) (Dojindo, Kumamoto, Japan). The absorbance was read at 450 nm using an ELISA reader (ELx800; Bio-Tek Instruments, Winooski, VT, USA).

### Apoptosis assay

AML cells infected with scramble or shMsi2 lentivirus were treated or untreated with insulin-like growth factor-1 (IGF-1) or thrombopoietin (TPO; both from PeproTech, Rocky Hill, NJ, USA) or daunorubin (Selleck Chemicals, Houston, TX, USA). Then, cells were harvested and analyzed for apoptosis using APC-Annexin V and propidium iodide (PI; BD Pharmingen™, San Diego, CA, USA) according to the manufacture’s protocols. Data acquisition and analysis were performed using CellQuest software on a flow cytometry (FACSCalibur; BD, Mountain View, CA, USA).

### Cell cycle assay

AML cells infected with scramble or shMsi2 lentivirus were harvested and stained with PI using a Cycletest™ plus DNA reagent kit (BD Biosciences, Franklin lakes, NJ, USA) according to the manufacturer’s instructions.

### Quantitative real-time PCR for gene expression analysis

Quantitative real-time PCR was used to analyze the expression of CCND1, p21, Cdk2, Bcl-2, Bax, and a reference gene GAPDH according to our previously reported method [19]. The sequences of specific primers see Table 2.

**Table 2:** The sequences of the primers used for real-time qPCR.

	Forward primer (5’-3’)	Reverse primer(5’-3’)
CCND1	CCCTCGGTGTCCTACTTCAA	AGGAAGCGGTCCAGGTAGTT
p21	GGAAGACCATGTGGACCTGT	GGCGTTTGGAGTGGTAGAAA
Cdk2	AAGATCGGAGAGGGCACGTA	CTCAGTCTCAGTGTCCAGGC
Bcl-2	ATGTGTGTGGAGAGCGTCAA	ACAGTTCCACAAAGGCATCC
Bax	AACATGGAGCTGCAGAGGAT	CAGTTGAAGTTGCCGTCAGA
GAPDH	ATCATCAGCAATGCCTCC	CATCACGCCACAGTTTCC

### Western blot assays

After infection with scramble or shMsi2 lentivirus, AML cells were collected and lysed immediately using M-PER^®^ Mammalian protein extraction reagent (Pierce, Rockford, IL, USA) supplemented with Halt protease and phosphatase inhibitor cocktail (Pierce). The protein was subjected to western blot analysis with antibodies against Msi2 (Abcam, Cambridge, UK), Cyclin D1, p21, Bcl-2, Bax, PARP, p-Akt (Ser473), Akt, p-Erk1/2 (Thr202/Tyr204), Erk1/2, p-p38 (Thr180/Tyr182), p38, or GAPDH (Cell Signaling Technology, Beverly, MA, USA) according to our previously described method [19,20]. The optical densities of the bands were analyzed using Image J software (NIH, Bethesda, MD, USA).

### In vivo experiments

NOD/SCID mice (SLAC Laboratory Animal Center, Shanghai, China), 6 weeks old, were maintained throughout in specific pathogen-free environment and divided into two groups of eight mice per group. A total of 1 × 10^7^ HL-60 cells expressing shMsi2-3 or scramble control were injected into the tail vein of each mouse. After transplantation, the mice were observed until they died of infiltration of HL-60 cells and the time of death was recorded. Animal procedures were carried out in accordance with institutional guidelines after Wenzhou Medical University and Fudan University Animal Care and Use Committee approved the study protocol.

### Statistical analysis

Data are expressed as mean ± SEM. Statistical analyses were performed using GraphPad Prism 5.0 (GraphPad software, San Diego, CA, USA). *P* values less than 0.05 was considered statistically significant.

## Results

### Msi2 expression in leukemic cell lines and primary AML cells

**Fig 1:**
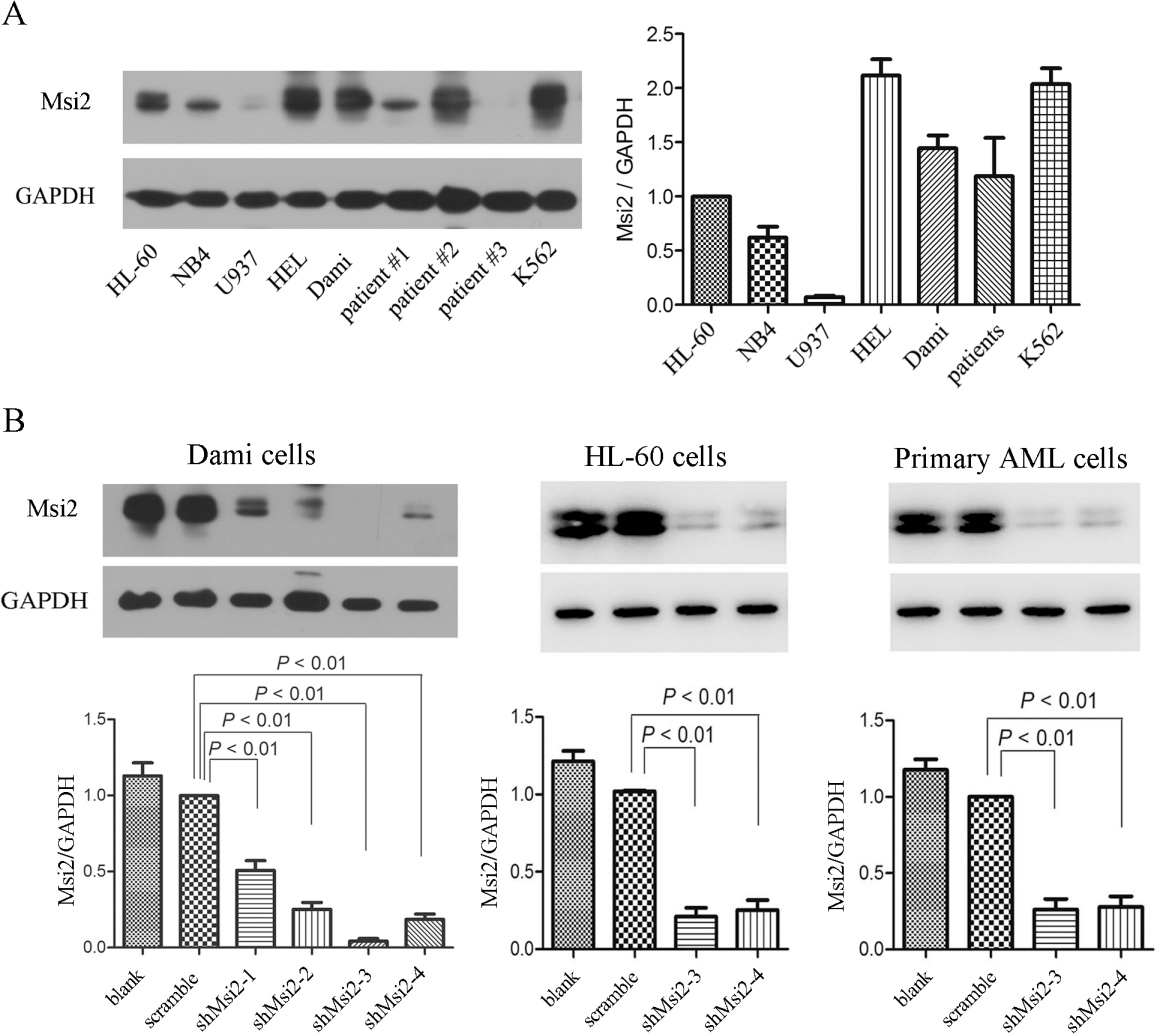
Msi2 expresses in leukemic cells and is down-regulated in AML cells. (**A**) The protein levels of Msi2 in leukemic cell lines and primary AML cells. Representatives and statistical data of 3 independent experiments demonstrating Msi2 expression in leukemic cell lines and primary AML cells from AML patients (n = 8) using western blot were shown. (**B**) Msi2 is down-regulated by lentivirus-mediated RNA interference in Dami cells, HL-60 cells, and primary AML cells. The levels of Msi2 were detected using western blot. Quantification of Msi2 normalized against GAPDH was shown (n = 3).

Msi2 expression was analyzed in five AML cell lines (HL-60, NB4, U937, HEL, and Dami) and primary AML cells from AML patients as well as a CML cell line K562. In contrast to HL-60 cells, high expression of MSI2 in HEL, Dami, and K562 cells, low expression of Msi2 in NB4 and U937 cells, and similar expression of Msi2 in primary AML cells are shown (Fig 1A). To identify the biological roles and explore the underlying mechanisms of Msi2 in AML cells, we chose two AML cell lines Dami and HL-60 as well as primary AML cells with high Msi2 expression to infect with lentivirus carrying shMsi2. Western blot analysis demonstrated that the expression of Msi2 was significantly decreased in four separate shMsi2 group in Dami cells, especially in shMsi2-3 and shMsi2-4 group (Fig 1B). Msi2 expression was also significantly decreased in HL-60 cells and primary AML cells infected with shMsi2-3 or shMsi2-4 lentivirus (Fig 1B).

### Msi2 silencing inhibits the proliferation of AML cells

**Fig 2:**
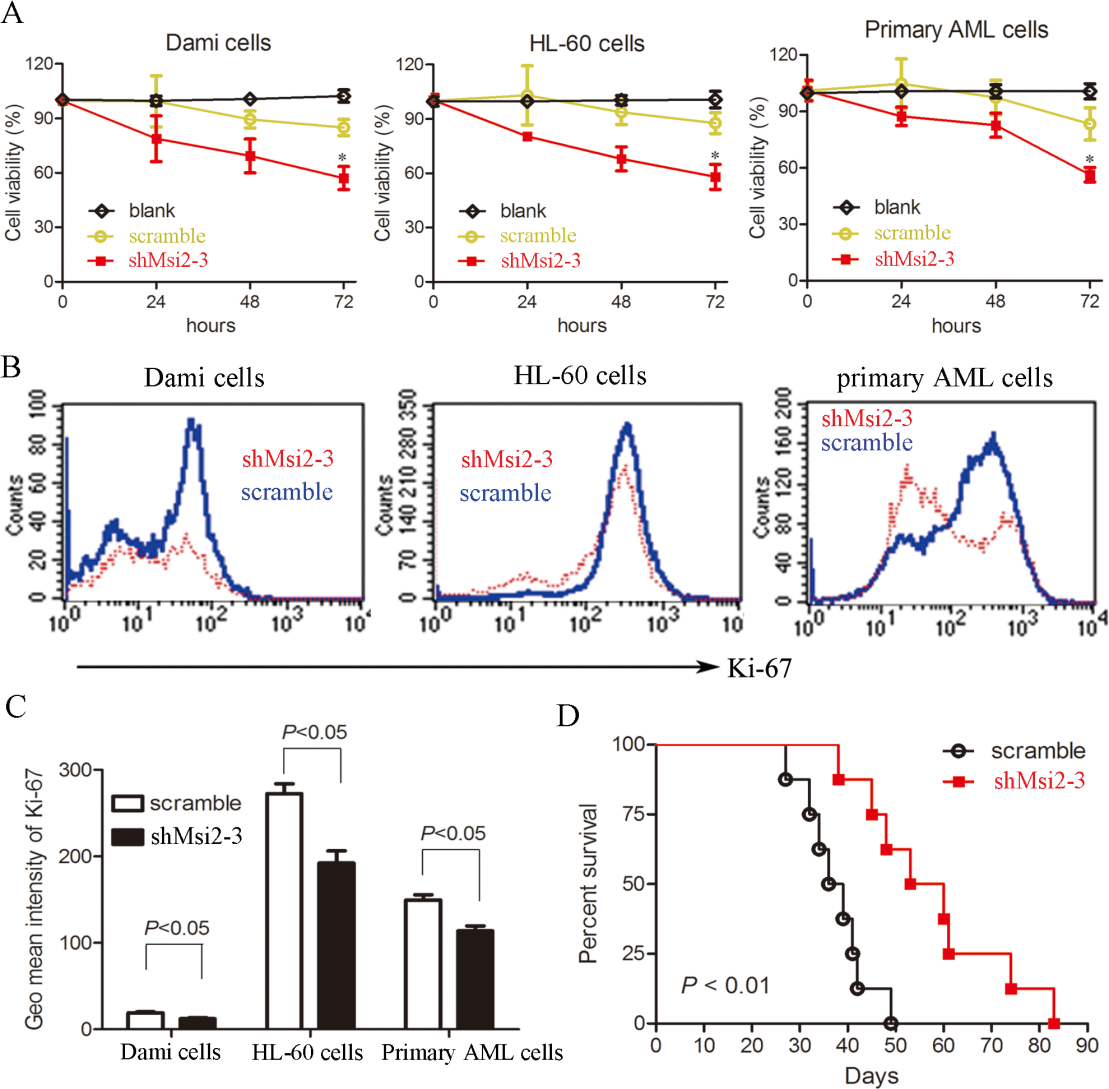
Msi2 silencing inhibits the proliferation of AML cells. (**A**) Msi2 silencing inhibits the proliferation of Dami cells, HL-60 cells, and primary AML cells from AML patients. Cells were plated at a density of 4 × 10^3^ cells/well and grown for 0 to 72 h, and cell viability was determined using CCK-8. (**B**)&(**C**) The Ki-67 expression was significantly decreased in shMsi2-3 group compared to scramble control group. Cells were harvested and fixed using 75% alcohol for 4 h at -20°C. Then, cells were washed and incubated with anti-Ki-67 Alexa Flour^®^647 antibody for 30 min at room temperature and finally determined using a flow cytometry. Representatives and statistical data from 3 independent experiments were shown. (**D**) NOD/SCID mice were intravenously injected with 1 × 10^7^ HL-60 cells infected with shMsi2-3 or scramble control lentivirus. Kaplan-Meier curves for overall survival were assessed and the Log-rank test was used to determine the statistical significance.

To determine whether Msi2 affects the proliferation of AML cells, cell viability assay was performed using CCK-8. The proliferation of AML cells including Dami cells, HL-60 cells, and primary AML cells were decreased in shMsi2-3 group compared to the scramble control group (Fig 2A). Msi2 silencing also significantly decreased the expression of Ki-67, a cell proliferation marker, in AML cells (Fig 2B and 2C). Furthermore, Msi2 silencing significantly prolonged the survival of NOD/SCID mice intravenously injected with HL-60 cells, indicating that Msi2 silencing inhibits the proliferation of HL-60 cells in vivo (Fig 2D). Taken together, these data indicate that Msi2 silencing suppresses the proliferation of AML cells.

### Msi2 silencing causes cell cycle arrest in AML cells

**Fig 3:**
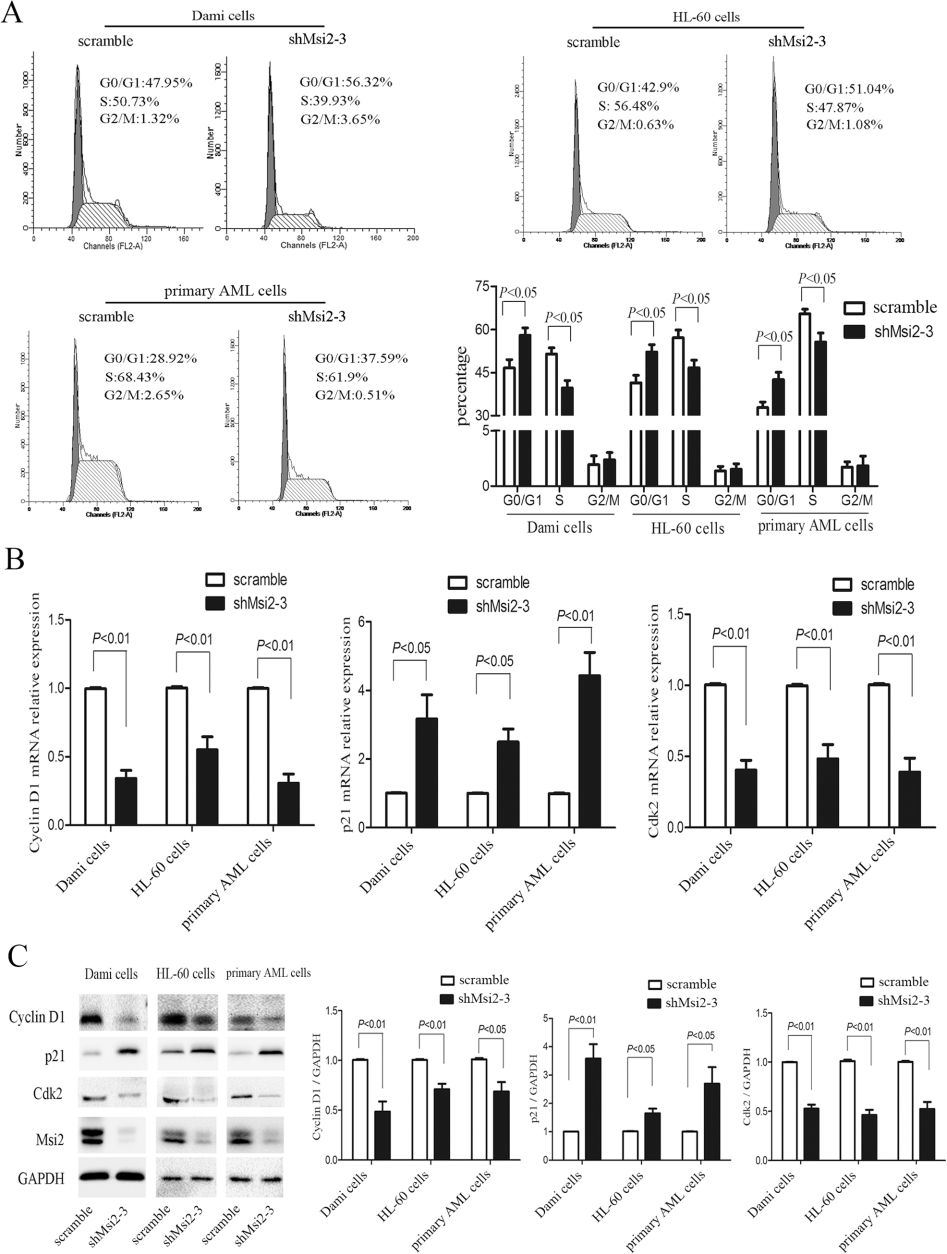
Msi2 silencing leads to G0/G1 cell arrest in AML cells. (**A**) Msi2 silencing increased the percentages of G0/G1 phase cells and decreased those of S phase cells in Dami cells, HL-60 cells, and primary AML cells from AML patients. Representatives and statistical data from 3 independent experiments were shown. (**B**) Msi2 silencing decreased the mRNA levels of CCND1 and Cdk2 and increased the mRNA level of p21 in AML cells. (**C**) Msi2 silencing decreased the protein levels of Cyclin D1 and Cdk2 increased the protein level of p21 in AML cells. Representatives and statistical data from 3 independent experiments were shown.

We next investigate whether Msi2 silencing affects the cell cycle progression of AML cells. As shown in Fig 3A, Msi2 silencing led to cell cycle arrest in G0/G1 phase, and subsequently decreased the fraction of S phase in Dami cells, HL-60 cells, and primary AML cells. We further analyzed the expression levels of Cyclin D1, p21, and Cdk2, three key G1 regulatory proteins [21,22]. Msi2 silencing significantly decreased the mRNA and protein levels of Cyclin D1 and Cdk2 and increased the mRNA and protein levels of p21 compared to the scramble group (Fig 3B and 3C). These data suggest that the G0/G1 arrest induced by Msi2 silencing in AML cells may be mediated by decreasing Cyclin D1 and Cdk2 expression and increasing p21 expression.

### Msi2 silencing induces apoptosis in AML cells

**Fig 4:**
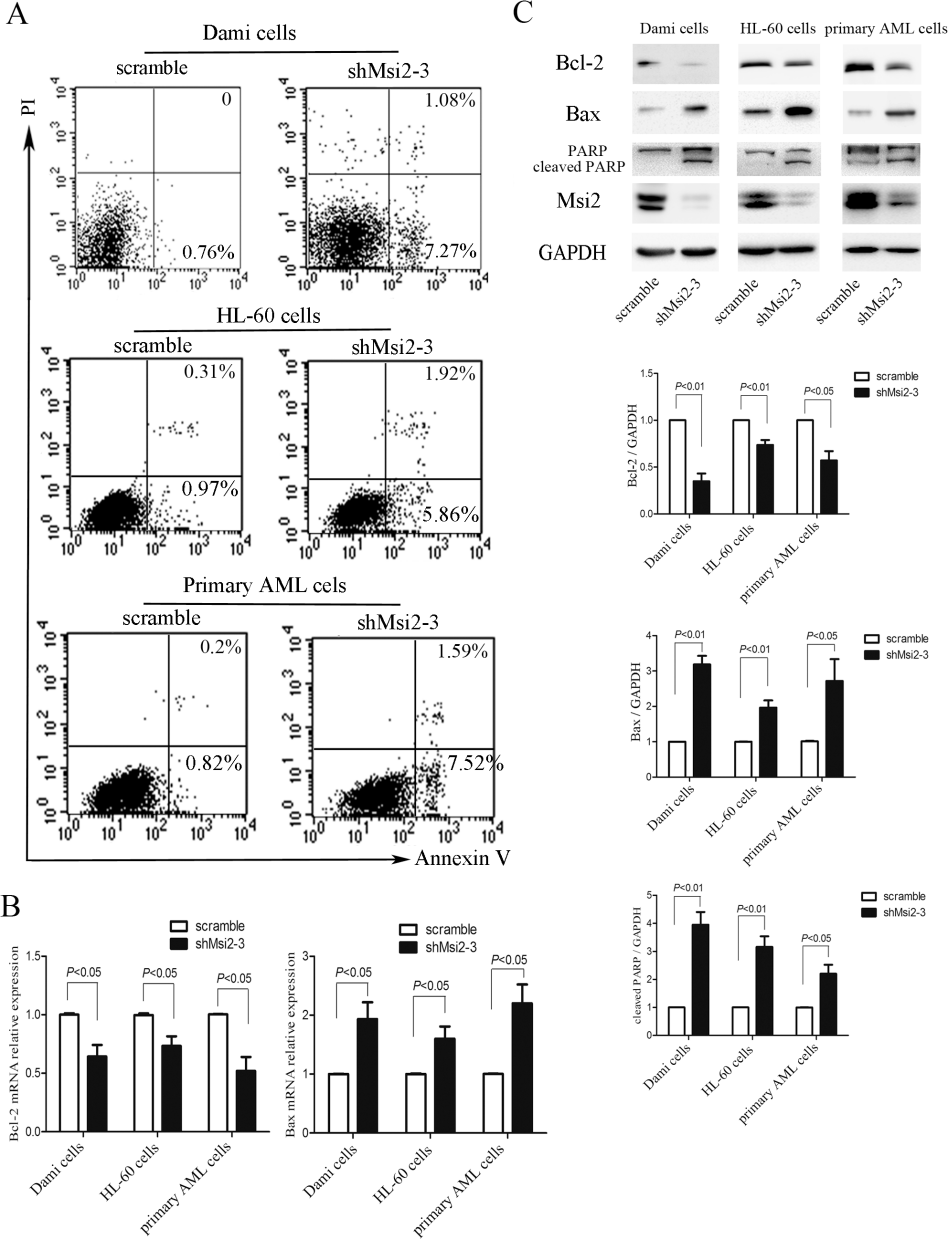
Msi2 silencing induces apoptosis in AML cells. (**A**) Msi2 silencing induces apoptosis in Dami cells, HL-60 cells, and primary AML cells from AML patients. The Annexin V-APC binding and PI staining method was used to assess apoptosis, and the results shown were representatives of 3 independent experiments. (**B**) Msi2 silencing decreased the mRNA level of Bcl-2 and increased the mRNA level of Bax in AML cells. (**C**) Msi2 silencing decreased Bcl-2 expression and increased Bax and cleaved PARP expression in AML cells using western blot. Representatives and quantification of Bcl-2, Bax or cleaved PARP normalized against GAPDH were shown (n = 3).

We then examined whether Msi2 silencing increases apoptosis in AML cells using flow cytometry analysis. Compared with the scramble control, apoptosis was significantly increased in shMsi2-3 group in Dami cells, HL-60 cells, and primary AML cells (Fig 4A), suggesting that Msi2 silencing in AML cells results in an accelerated apoptosis. Bcl-2 is a pivotal anti-apoptotic effector protein recently proposed to play a crucial role for the propagation of AML [23], and Bax, a key pro-apoptotic effector protein, plays a key role in promoting apoptosis [24]. We next investigate whether these two proteins are involved in shMsi2-mediated apoptosis. As shown in Fig 4B and 4C, the mRNA and protein levels of Bcl-2 were significantly decreased in shMsi2-3 group compared to the scramble control group, while the mRNA and protein levels of Bax were markedly increased relative to the scramble control group. We also determined the level of cleaved PARP, an apoptosis-related protein, and found that Msi2 silencing did increase the levels of cleaved PARP in AML cells (Fig 4C). Taken together, Msi2 silencing induced a modest, but significant apoptosis in AML cells compared with the scramble control.

### Msi2 silencing inhibits the phosphorylation of Akt, Erk1/2 and p38

**Fig 5:**
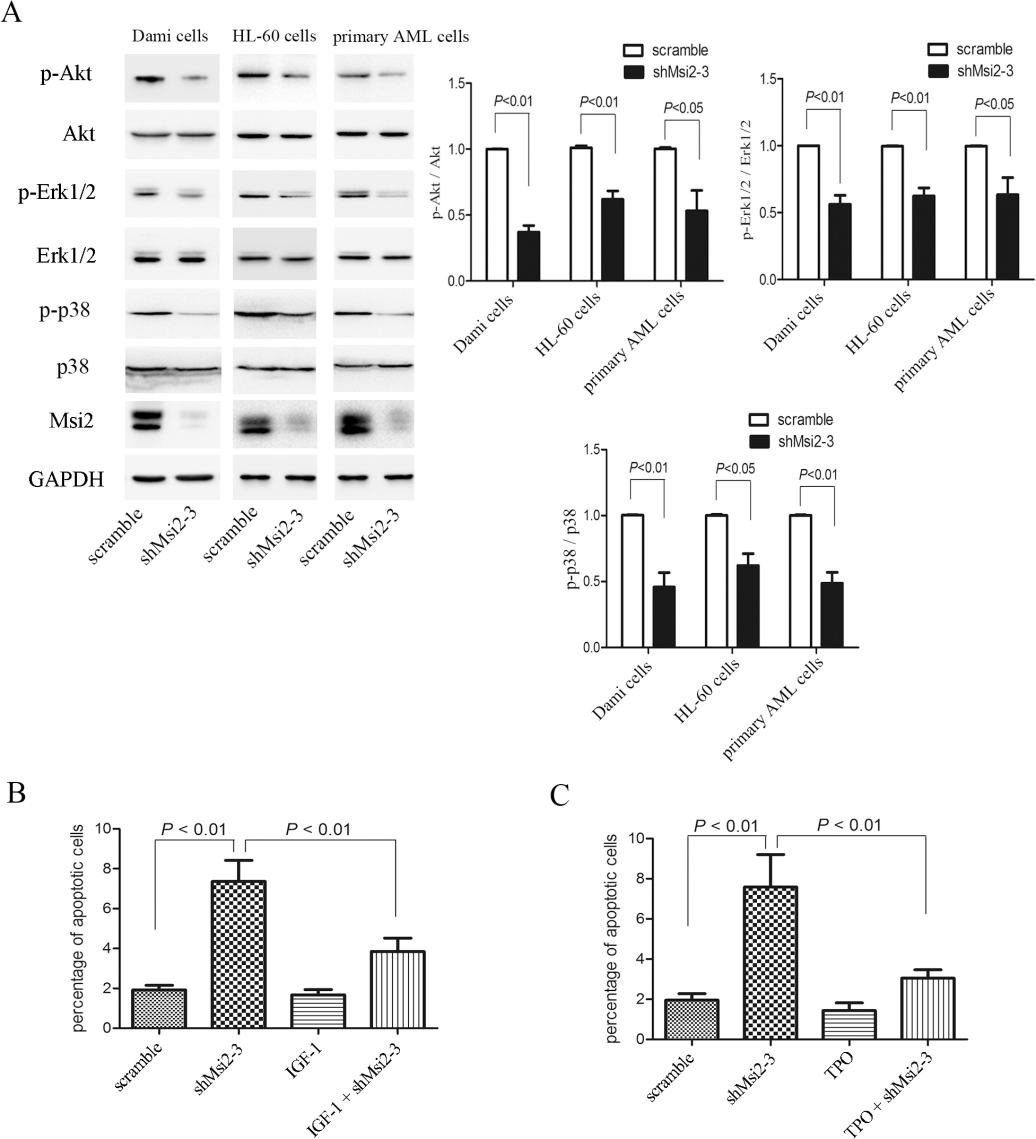
Msi2 silencing inhibits Akt, Erk1/2 and p38 signaling, which contributes to apoptosis mediated by Msi2 silencing in AML cells. (**A**) The phosphorylation of Akt, Erk1/2 and p38 were decreased followed by Msi2 silencing in Dami cells, HL-60 cells and primary AML cells from AML patients. Representatives and statistical data of 3 independent experiments were shown. (**B**) IGF-1 attenuated Msi2 silencing-induced apoptosis in Dami cells. Apoptotic cells were determined using Annexin V/PI double staining method in the presence or absence of IGF-1 (100 ng/ml) for 24 h (n = 3). (**C**) TPO attenuated Msi2 silencing-mediated apoptosis in Dami cells. Apoptotic cells were determined using Annexin V/PI double staining method with or without TPO (100 ng/ml) for 2 h (n = 3).

To further determine the mechanisms regulated by Msi2 in the induction of apoptosis in AML cells, the phosphorylation of Akt, Erk1/2 and p38 were examined. Msi2 silencing decreased the phosphorylation of Akt in Dami cells, HL-60 cells, and primary AML cells (Fig 5A), inconsistent with a previous report in which Msi2 silencing did not affect the phosphorylation of Akt in K562 cells [25]. To further confirm whether Akt participates in apoptosis mediated by Msi silencing, IGF-1, a potent activator of the PI3K/Akt signaling pathway [26], were used. As shown in Fig 5B, IGF-1 significantly inhibited shMsi2-mediated apoptosis of Dami cells, indicating that the Akt signaling is involved in apoptosis mediated by Msi2 silencing in AML cells. MSI2 silencing also decreased the phosphorylation of Erk1/2 and p38 in Dami cells, HL-60 cells, and primary AML cells (Fig 5A). TPO, a strong activator of Erk1/2 signaling [27], inhibited shMsi2-mediated apoptosis of Dami cells (Fig 5C). These data suggest that induction of apoptosis by Msi2 silencing in AML cells may be mediated by inhibition of Akt, Erk1/2, and p38 signaling.

### Msi2 silencing enhances chemosensitivity of AML cells to daunorubicin

**Fig 6:**
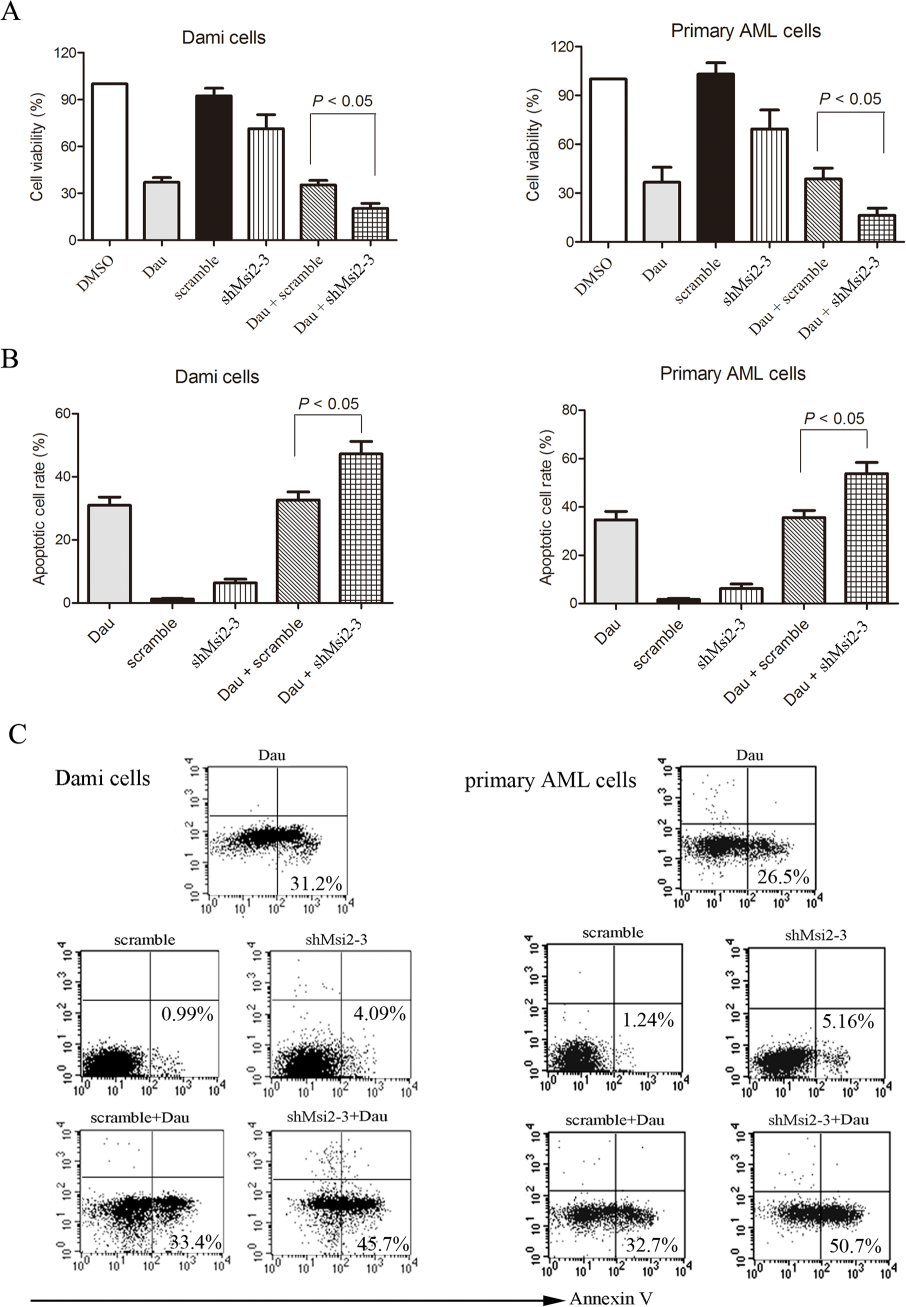
Msi2 silencing enhances chemosensitivity of AML cells to daunorubicin. Dami cells and primary AML cells isolated from AML patients infected with shMsi2-3 or scarmble lentivirus were untreated or treated with 200 nM daunorubicin for 48 h and cell viability (**A**) was measured using CCK-8. Data are expressed as mean ± SEM representing 3 independent experiments. Meanwhile, apoptosis was measured using Annexin V staining. Statistical data (**B**) and representatives (**C**) of 3 independent experiments were shown.

Based on abovementioned findings, we then investigated whether AML cells with reduced Msi2 expression are more sensitive to daunorubicin, an anthracycline in combination with cytarabine as the standard induction therapy for AML patients of all subtypes except M3 [28]. As shown in Fig 6A, Msi2 silencing exhibited markedly higher proliferation inhibitory rates compared to the scramble control in combination with daunorubicin for 48 h in Dami cells and primary AML cells. In addition, we examined whether Msi2 silencing enhances daunorubicin-induced cell apoptosis. As shown in Fig 6B and 6C, Msi2 silencing in combination with daunorubicin resulted in a marked increase in apoptosis compared to the scramble control group in combination with daunorubicin, suggesting that Msi2 silencing sensitizes AML cells to daunorubicin.

## Discussion

Increasing evidences have shown that Msi2 overexpression is a common characteristic of leukemic cells, where up-regulation of Msi2 was negatively associated with rapid progression and poor prognosis. However, the biological roles and the underlying mechanisms mediated by Msi2 in AML are not well understood. Here we found that Msi2 silencing inhibited proliferation and induced apoptosis and enhanced chemosensitivity to daunorubicin in AML cells. In addition, we also found that Akt, Erk1/2 and p38 signaling were involved in apoptosis induced by Msi2 silencing in AML cells.

We measured the expression of Msi2 in five AML cell lines and a CML cell line K562 as well as primary AML cells isolated from AML patients. High Msi2 levels were observed in HEL and Dami cells, whereas low Msi2 levels were observed in NB4 and U937 cells among five AML cell lines, similar to a previous report [25]. The analysis of Msi2 protein expression in primary AML cells showed great heterogeneity, similar to the transcript levels previously reported [14]. AML cells including Dami cells, HL-60 cells and primary AML cells were infected with lentivirus carrying shMsi2 and successful inhibition of Msi2 expression was observed as evidenced by a significant decrease of Msi2 using western blot analysis.

Cell viability assay demonstrated that knockdown of Msi2 inhibited the proliferation of Dami cells, HL-60 cells, and primary AML cells. Consistent with our results, Msi2 silencing also resulted in decreased proliferation in other AML cell lines and a CML cell line [11,25]. The reduced Ki-67 expression mediated by Msi2 silencing further confirmed that Msi2 contributed to leukemogenesis. Msi2 silencing also significantly suppressed HL-60 cell growth and prolonged survival in a NOD/SCID mice model. Recently, many studies have indicated that the cell growth inhibition is mainly caused by cell cycle arrest and/or apoptosis [18,29,30]. In this study, we found that Msi2 silencing resulted in cell cycle arrest in G0/G1 phase and reduced the percentage of cells in S phase in AML cells. Cyclin D1 and p21 are two classical cell cycle-related proteins. We found that Msi2 silencing in AML cells decreased Cyclin D1 expression and increased p21 expression, consistent with the observed G0/G1 cell cycle arrest. Therefore, cell cycle arrest may be one of the causes whereby Msi2 silencing leads to inhibition of proliferation in AML cells.

In addition to cell cycle arrest, we also found that Msi2 silencing in AML cells markedly increased apoptotic cells compared to the scramble controls. Western blot analysis demonstrated that Msi2 silencing significantly decreased anti-apoptotic protein Bcl-2 and increased pro-apoptotic protein Bax expression, implying the role of Msi2 as a regulator of apoptosis. The PI3K/Akt pathway plays important roles in leukemic cell proliferation, growth, and survival [18,31]. We found that Msi2 silencing resulted in a significant decrease in Akt phosphorylation. IGF-1 reduced apoptosis induced by Msi2 silencing, which further confirmed that the PI3K/Akt pathway is involved in Msi2-mediated leukemogenesis. The MAPK pathway also plays a critical role in the regulation of diverse cellular processes, such as proliferation, apoptosis, differentiation, and inflammation [32]. It has been reported that Msi2 silencing leads to the inactivation of MAPK pathway in K562 cells [25]. Similarly, we also found that Msi2 silencing decreased the phosphorylation of Erk1/2 and p38 in Dami cells, HL-60 cells, and primary AML cells. TPO treatment reduced apoptosis induced by Msi2 silencing in Dami cells, which also further confirmed that the Erk1/2 signaling was downstream of Msi2.

Anthracycline antibiotics, such as daunorubicin, are still key chemotherapeutic drugs used in AML treatment [33]. In this study, we showed that the cytotoxicity of daunorubicin was significantly enhanced by Msi2 silencing, and the combination of daunorubicin and Msi2 silencing had greater antiproliferative effects than daunorubicin treatment alone, suggesting that Msi2 may be involved in chemoresistance in AML cells. Furthermore, our results also showed that combination of daunorubicin treatment and Msi2 silencing significantly increased apoptosis. Taken together, these findings suggest that the combination of gene therapy with conventional chemotherapeutics may be an attractive therapeutic strategy in leukemia treatment.

In conclusion, this study demonstrated that Msi2 silencing exerted potent activity against AML and enhanced the cytotoxicity of daunorubicin. Therefore, Msi2 may be a promising therapeutic target for AML.

## Supporting Information

S1 ARRIVE Checklist The ARRIVE Guidelines Checklist. (PDF)
